# Aberrant Right Subclavian Artery Causing Dysphagia: A Case Report of Dysphagia Lusoria

**DOI:** 10.7759/cureus.25980

**Published:** 2022-06-15

**Authors:** Mahmoud M Mansour, Mohammad Darweesh, Ratib Mahfouz, Adham E Obeidat, Kanak Das

**Affiliations:** 1 Internal Medicine, University of Missouri School of Medicine, Columbia, USA; 2 Internal Medicine, East Tennessee State University, Johnson City, USA; 3 Internal Medicine, Kent Hospital/Brown University, Warwick, USA; 4 Internal Medicine, University of Hawaii, Honolulu, USA; 5 Gastroenterology and Hepatology, University Medical Center, Lubbock, USA

**Keywords:** vascular anomaly, mechanical dysphagia, dysphagia, aberrant right subclavian artery, dysphagia lusoria

## Abstract

Dysphagia lusoria is a rare condition, with a prevalence of less than 1%, that occurs through secondary compression of the esophagus posteriorly by an aberrant right subclavian artery. It commonly presents with dysphagia to solids. Management is usually done with dietary modification; however, more severe and intractable cases may require surgical intervention. We describe this rare vascular anomaly in a 54-year-old female presenting with mechanical dysphagia.

## Introduction

Dysphagia lusoria is a rare condition characterized by swallowing impairment secondary to extrinsic compression of the esophagus by an aberrant right subclavian artery. The aberrant right subclavian artery is the most common congenital anomaly of the aortic arch with a prevalence of 0.16%-4.4% [1]. Instead of being a branch of the brachiocephalic artery along with the right common carotid artery, the aberrant right subclavian artery arises on its own distal to the left subclavian artery. It then takes a course posterior to the esophagus to reach the right side [1]. This anomaly is symptomatic in about 30% of patients, and dysphagia, the most common presentation, is usually consistent with mechanical obstruction [2].

This article was previously presented as a meeting abstract at the 2021 ACG Annual Meeting on October 25, 2021.

## Case presentation

A 54-year-old female with a medical history of hypothyroidism presented to the gastroenterology clinic with progressively worsening difficulty swallowing for the past six months. The patient reported a sensation of "food getting stuck in her upper chest," episodes of regurgitation of undigested food, and chronic heartburn along with eight pounds of unintentional weight loss over six months. Her physical exam and laboratory studies were unremarkable.

**Figure 1:**
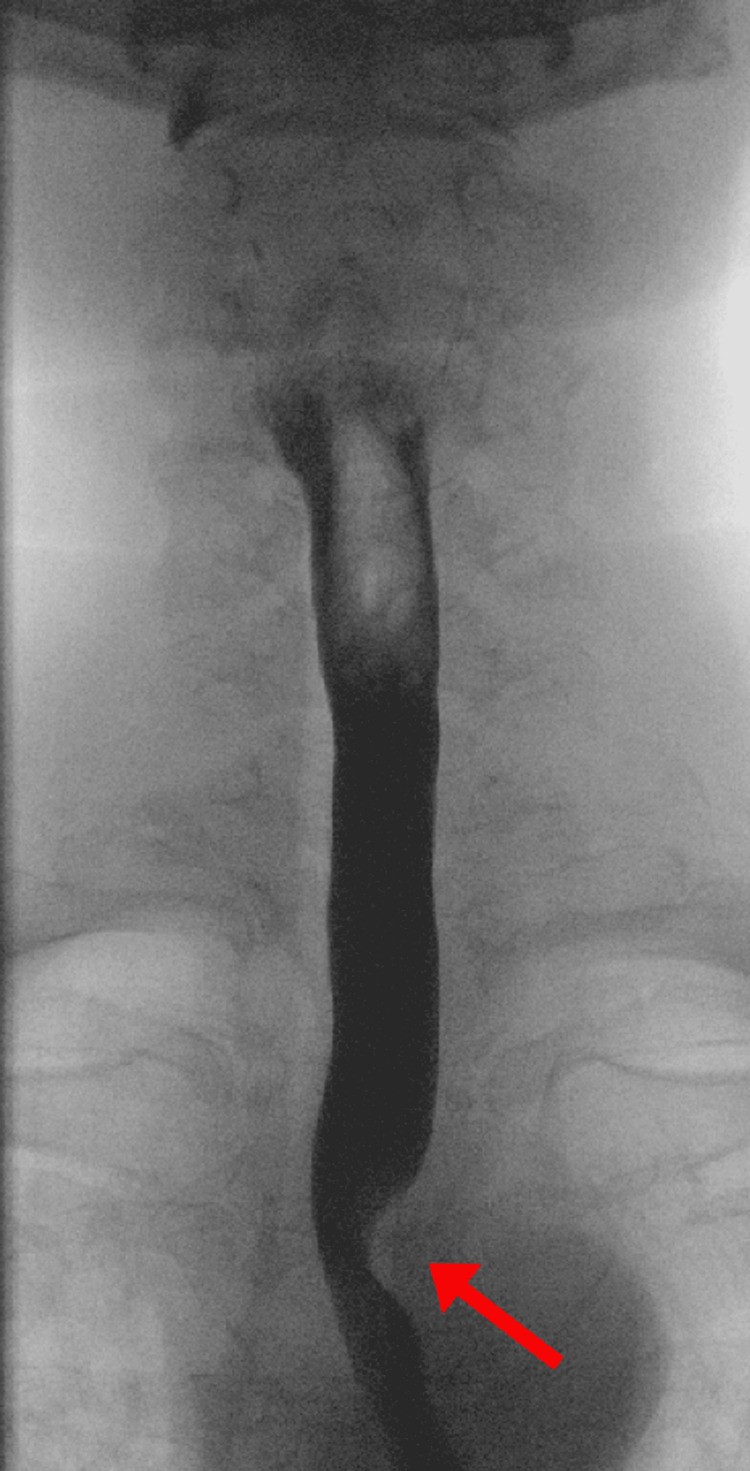
Barium esophagogram showing extrinsic impression on the left aspect of the thoracic esophagus (red arrow)

**Figure 2:**
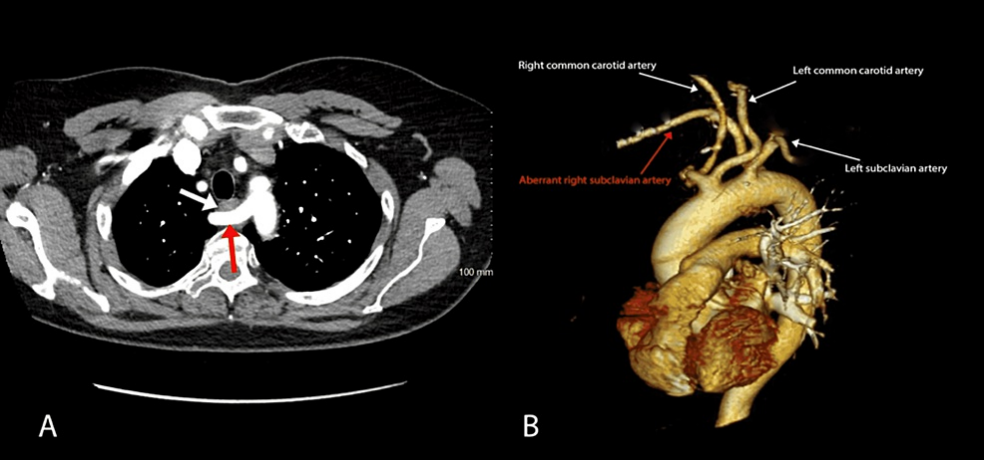
(A) CT angiography scan showing aberrant right subclavian artery (red arrow) coursing posterior to the esophagus (white arrow), resulting in extrinsic compression. (B) Three-dimensional CT scan showing the heart and aortic arch. The right subclavian artery (highlighted in red) arises as the last branch from the aortic arch distal to the origin of the left subclavian artery.

**Figure 3:**
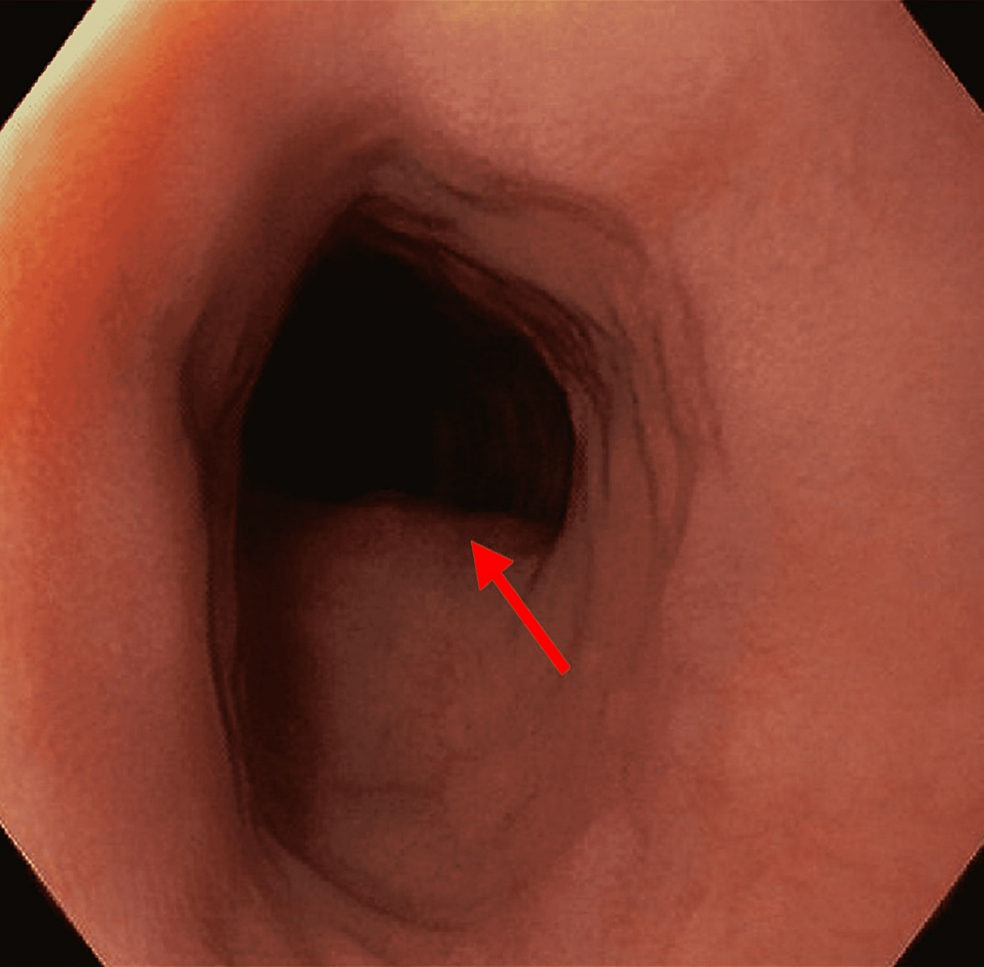
EGD showing external compression (red arrow) posterior to the esophagus EGD: Esophagogastroduodenoscopy.

A barium esophagogram demonstrated extrinsic compression on the left aspect of the esophagus just superior to the aortic arch (Figure 1), concerning an aberrant right subclavian artery compressing the esophagus. Subsequent computed tomography (CT) angiogram confirmed that the artery was compressing the esophagus as it traversed it posteriorly, anterior to the vertebral column as shown in Figure 2 (Panels A and B). In addition, esophagogastroduodenoscopy (EGD) evaluating dysphagia and chronic heartburn demonstrated mild esophagitis and a pulsating oblique protrusion in the left posterior wall of the upper esophagus (Figure 3). These findings were consistent with a diagnosis of dysphagia lusoria.

Although heartburn was controlled with proton pump inhibitor therapy, the dysphagia did not improve with dietary modifications over a four-month treatment period. Consequently, after discussions with the vascular surgery team, the patient opted for vascular reconstruction in which a subclavian bypass with interval ligation via a thoracoabdominal approach was performed to alleviate the esophageal compression. Postoperatively, recovery was uneventful, and the patient was discharged on postoperative day five. Six months following surgery, the patient reported no symptoms of dysphagia and no complications related to surgery.

## Discussion

In dysphagia lusoria, the aberrant right subclavian artery assumes a retro-esophageal course compressing the esophagus [1]. It commonly presents during the fourth and fifth decades of life, which is possibly due to atherosclerotic hardening or fibromuscular dysplasia of the arteries with advancing age [3]. Other less common manifestations of an aberrant right subclavian artery are related to tracheal compression and include dyspnea and chronic cough [3].

The diagnosis of dysphagia lusoria can be established by a barium esophagogram followed by CT angiography or magnetic resonance (MR) angiography to define the vascular anatomy [4]. An upper endoscopy can demonstrate a pulsating impression on the esophagus posteriorly, though this finding can often be missed. Manometry is usually not helpful in diagnosis but can be considered to evaluate associated esophageal dysmotility [5].

The management of dysphagia lusoria primarily depends on the severity of symptoms and the impact on nutrition and weight. Less severe cases can be treated with dietary modification, such as eating smaller bites, sipping liquids, and eating slower [3]. Additionally, treatment of associated conditions such as gastroesophageal reflux disease and esophageal dysmotility should be considered [6]. Surgical interventions with the reconstruction of the aberrant vessel may be necessary for severe or intractable cases [6].

## Conclusions

This case highlights the rare condition of dysphagia lusoria where an anomalous right subclavian artery creates a vascular sling that compresses the esophagus. A high index of suspicion is essential for obtaining the proper diagnostic workup with the appropriate imaging to detect the vascular abnormality. Patients with severe symptoms or those who failed to respond to medical management can be considered for surgical interventions to reposition the aberrant vessel.
